# Head injury on Warfarin: likelihood of delayed intracranial bleeding in patients with negative initial head CT

**DOI:** 10.1186/s13104-018-3291-z

**Published:** 2018-03-15

**Authors:** Amer Afaneh, Jennifer Ford, Jenna Gharzeddine, Alexandre Mazar, R. David Hayward, Joseph Buck

**Affiliations:** Department of Surgery, Division of Trauma, St. John Hospital & Medical Center, 22151 Moross Rd., PB I, Suite 212, Detroit, MI 48236 USA

**Keywords:** Traumatic injury, Warfarin, Intracranial bleeding, Head CT

## Abstract

**Objective:**

To determine the likelihood that head injured patients on Warfarin with a negative initial head CT will have a positive repeat head CT. A retrospective chart review of our institution’s trauma registry was performed for all patients admitted for blunt head trauma and on Warfarin anti-coagulation from January 2009 to April 2014. Inclusion criteria included patients over 18 years of age with initial GCS ≥ 13, INR greater than 1.5 and negative initial head CT. Initial CT findings, repeat CT findings and INR were recorded. Interventions performed on patients with a delayed bleed were also investigated.

**Results:**

394 patients met the study inclusion criteria. 121 (31%) of these patients did not receive a second CT while 273 patients (69%) underwent a second CT. The mean INR was 2.74. Six patients developed a delayed bleed, of which two were clinically significant. No patients had any neurosurgical intervention. Our results demonstrate a low rate of delayed bleeding. The utility of repeat head CT in the neurologically stable patient is thus questioned. Patients who have an abnormal baseline neurological status and those with INR >3 may represent a subgroup of patients in whom repeat head CT should be performed.

## Introduction

**Fig. 1:**
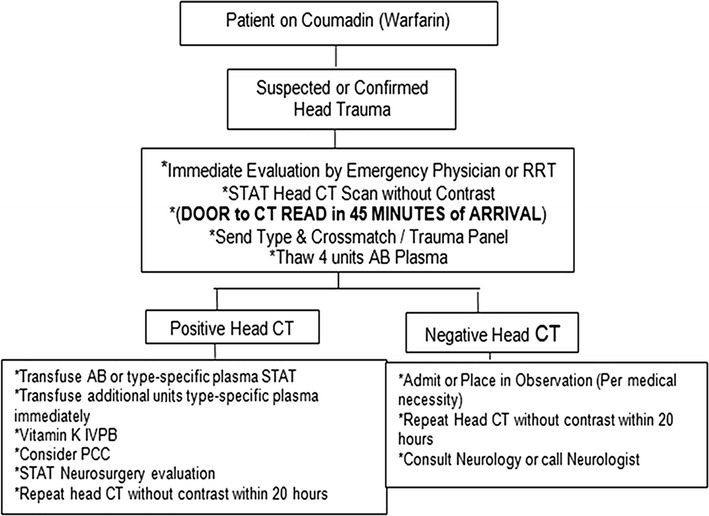
Institutional adult head injury on warfarin protocol flowchart, beginning July 2011 (N = 555)

Emergency physicians, internists and surgeons frequently see the complications of anticoagulation [1]. In a trauma case, a patient taking Warfarin who has a head injury will likely receive head X-ray computed tomography (CT) to rule out intracranial bleeding [2, 3]. However, there is as yet limited consensus regarding criteria for determining which patients should receive additional observation in these circumstances [4]. At our institution, the “Head Injury on Warfarin Protocol” calls for an immediate head CT, as well as an optional repeat head CT within 20 h (Fig. 1). A patient with a head injury and a negative initial CT is typically admitted or observed and receives a repeat CT head the following morning. The purpose of the repeat CT is to rule out delayed intracranial bleeding. In this study, we investigated the outcomes of these anti-coagulated patients with head injury who have a negative initial head CT and to determine the likelihood of delayed intracranial hemorrhage.

## Main text

### Methods

This study was approved by the Institutional Review Board of St. John Hospital & Medical Center (Detroit, Michigan, USA), which granted approval for a waiver of consent (protocol number SJ0914-02). A retrospective chart review was performed for all patients who presented to St. John Hospital & Medical Center and were admitted or placed in Observation for “Fall on Warfarin” from January 1, 2009 to August 2014. The institution’s trauma registry was accessed for study information. Background information was collected including age and gender, as well as elements of injury severity (GCS and INR).

Patients included in the study were those suffering from head trauma and currently using Warfarin who were at least 18 years of age, with an international normalized ratio (INR) > 1.5, GCS between 13 and 15, and initial head CT that was negative for intracranial bleeding. Exclusion criteria included positive initial CT, normal or unavailable initial INR, and unavailable GCS data on admission.

Initial CT findings, repeat CT findings and INR were recorded. The results of the neurological exam (if available) and Glasgow Coma Scale (GCS) were recorded. Both initial and repeat CTs were coded as either negative or positive by the radiologist. Positive CT was indicated by the documented presence of an acute intracranial hemorrhage such as subarachnoid hemorrhage, intraparenchymal hemorrhage, subdural hematoma or epidural hematoma. Because the study was conducted retrospectively, the radiologists were blind to its hypotheses. Comparison between repeat and initial CT was based on match between determination as negative or positive. Analyses were conducted using SAS 9.4.

### Results

**Fig. 2:**
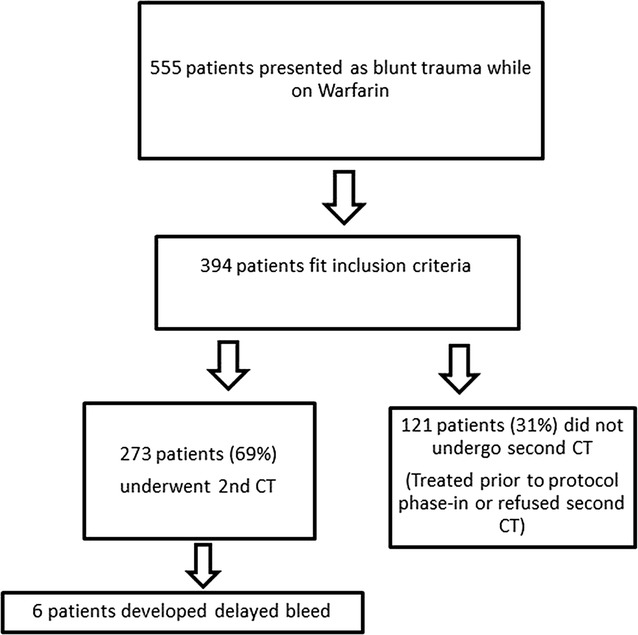
Flowchart for adult head injury patients on warfarin during study period (January, 2009–August, 2014) and negative initial CT with outcomes of repeat CT (N = 555)

Our trauma registry contained 555 patients who presented during our study period with blunt head injury and on Warfarin therapy (see Fig. 2). Three hundred ninety-four patients fit the inclusion criteria for our study. One hundred twenty-one (31%) of these patients did not receive a second CT while 273 patients (69%) underwent a second CT. Most of the patients who did not undergo a second CT were treated prior to the full phase-in of the current protocol for head injury on Warfarin at our institution, which began in October 2011, before which time the decision to order a second CT depended upon surgeon preference. These patients were discharged without observation.

Descriptive statistics are reported in Table 1. One hundred seventy-eight (45%) patients were male and 216 (55%) were female. The mean age was 74.19 ± 12 with a range between 22 and 97. The mean INR was 2.74 with a range between 1.5 and 22.36. The mean GCS was 14.84 ± 1.1. Of the patients who received a second head CT, 6 developed a delayed bleed (2.2%). These patients had a mean INR of 3.25. None of these patients had any neurosurgical intervention. Two out of the 6 patients had a clinically significant bleed, as defined by bleeds requiring clinical treatment.

**Table 1:** Descriptive statistics (N = 555)

	Range	Mean (SD) or N (%)
Age	22–97	74.19 (12.0)
Sex		
Male		175 (44.8%)
Female		216 (55.2%)
GCS	13–15	14. 84 (1.1)
INR	1.50–22.36	2.74 (5.2)
Repeat CT		273 (69.8%)

### Discussion

Six of the 273 patients who had a repeat head CT (2.2%) developed a delayed bleed. Only two patients had a clinically significant bleed (0.7%). A chart review of the six patients who had a delayed bleed did not reveal that they had any neurosurgical intervention. Of the two patients that had a clinically significant bleed, one patient was transferred to a rehabilitation facility due to overall debility. The second patient had neurologic decline and was ventilator dependent. The patient had multiple injuries and comorbidities and was given palliative care. It was unclear on examination of the medical record if neurologic changes preceded the repeat CT or if they occurred after the repeat CT. The mean INR of the six patients with delayed bleed was 3.25, which was greater than the mean INR for the entire group (2.74). Patients presenting with INR greater than 3 may represent a subset of patients who may have increased benefit of a repeat CT. Because the GCS range of the sample was restricted to between 13 and 15, there was not sufficient variance in this measure to draw useful conclusions regarding its relationship with CT outcomes.

Interpreting these results in the context of the costs and risks associated with administering repeated CT scans. CT scans are expensive and expose the patient to radiation. At our hospital, for example, the charge of a CT scan of the brain for a patient is approximately $1100 (this figure is derived from current hospital charges, and is not based on charge data for patients in this study). The cost of an unenhanced CT of the brain varies widely however and depending on insurance the amount the hospital actually receives is variable. This number however was used to perform a cost analysis. In order to detect two clinically significant delayed bleeds, 273 repeat CTs were performed. That comes to a total charge amount of more than $300,300. In the case of our two patients, no additional intervention was undertaken which raises the question of the cost utility of ordering these repeat scans. Additionally, all patients were exposed to potentially unnecessary doses of radiation resulting from the repeated CT.

Our study is consistent with the findings in the literature of low rate of delayed bleeding in patients on warfarin. Peck et. al found a 1% delayed hemorrhage rate however the study looked at both Warfarin and patients on anti-platelet therapy [5]. In a study comparing immediate and delayed hemorrhage in patients with pre-injury Warfarin or Clopidogrel use, Nishijima et. al found a 0.6% rate of delayed hemorrhage in patients on Warfarin [6]. The highest rate of delayed bleeding that we found in a literature search was 6% [7]. This was a prospective study in which 87 patients with minor head injury were observed and a negative initial CT was followed by a repeat CT in 24 h. They found that an INR greater than 3 represented a potential risk factor for delayed bleeding.

### Conclusions

The goal of our study was to look at the rate of delayed bleeding in patients on warfarin anticoagulation. Our results demonstrate a low rate of delayed bleeding in warfarin anticoagulated patients presenting after head injury. The utility of repeat head CT in the neurologically stable patient is thus questioned and may not be necessary in all patients. We believe repeat head CT may be beneficial in patients with baseline abnormal neurologic status and those with INR greater than 3.

## Limitations

Limitations of our study include those inherent to a retrospective study. Thirty-one percent of the patients (121/394) that fit our inclusion criteria did not undergo a repeat head CT. The reason for some patients not receiving a second CT scan is believed to be secondary to surgeon preference at the time. The protocol at our institution was established in 2011 and prior to and around that time, surgeon preference played a role in whether patients received a second CT. This may have skewed the estimate of the delayed bleeding rate. However, since it is likely that surgeons would have tended to send the patients they judged to have the highest risk to observation, and discharged those with lower risk, this design issue is more likely to lead to an overestimate of the overall rate of delayed bleeding, rather than an underestimate.

## Abbreviations

CT: X-ray computed tomography
GCS: Glasgow Coma Scale
INR: international normalized ratio
